# Social learning-based health literacy promotion on the self efficacy and social anxiety of adolescents with type 1 diabetes

**DOI:** 10.1186/s40842-024-00167-8

**Published:** 2024-03-15

**Authors:** Jamalodin Begjani, Akram Sadat Sadat Hosseini, Hedieh Saneifard, Vida Rahimi Hasanabad

**Affiliations:** 1 Tehran University of Medical Sciences, Tehran, Iran; 2 Shahid Beheshti University of Medical Sciences, Tehran, Iran

**Keywords:** Bandura’s social learning theory, Health literacy, Self-efficacy, Social anxiety, Adolescents, Type 1 diabetes

## Abstract

**Background and objective:**

Type 1 diabetes mellitus one of the biggest health concerns around the world, is difficult to manage during adolescence. Among the non-medical methods of controlling this disease is empowerment through self-efficacy. Poor self-efficacy leads to social anxiety and ultimately deficiencies in diabetes. There is also a correlation among health literacy, self-efficacy, and social anxiety. Thus, the present study aimed to evaluate the impact of a literacy promotion training program based on social learning theory on the self-efficacy and social anxiety of adolescents with T1DM.

**Methods:**

The current research is a semi-experimental type that was carried out with the cooperation of 66 adolescents aged 15–18 years with type 1 diabetes in Iran (Tehran, 2022). It has control and intervention groups. The endocrinology and diabetes clinics of the intervention and control groups were randomly selected in a multi-stage manner (endocrine and diabetes clinic of children’s medical center hospital for the control group and endocrine and diabetes clinic of Mofid hospital for the intervention group) and the participants were selected by Simple Random Sampling method (draw). The training program designed based on Bandura’s social learning theory was used to teach adolescents during seven consecutive sessions of 30–45 min during one week. Questionnaires were completed before and one month after the intervention. Data were analysed in SPSS-25 software.

**Findings:**

The intervention for adolescents with T1DM in intervention group compared to the control group had a significant effect on improve health literacy (*P*<0.001), self-efficacy (*P*<0.001), and social anxiety (*P*<0.05).

**Conclusions:**

The results can also be used to improve the capabilities of adolescents with T1DM, reduce and prevent disease complications, and develop operational-educational programs in the centers from which these adolescents receive various services.

**Trial registration:**

IRCT20210422051045N1.

## Background

Diabetes, which is one of the most common chronic diseases and the biggest health problem worldwide, has been called a silent epidemic by the WHO [1, 2]. Type 1 diabetes mellitus (T1DM) can occur at any age, but it is more common in childhood and adolescence [3, 4]. According to 2020 surveys, the prevalence of type 1 diabetes in the world, Asia, Africa, Europe, and America is 9.5, 6.9, 3.5, 12.2, and 12.2 per 10,000 people, respectively [5]. Currently, the number of people with T1DM in Iran is 11.4% [6]. Of every 400–500 children, one develops T1DM and is responsible for 6.7 million deaths in 2021, 1 death every 5 s and number of children and adolescents with type 1 diabetes is 1.2 million [7]. The importance of the problem becomes clearer when we know that one out of every ten Iranian adolescents has T1DM [8, 9]. Adolescence is considered a challenge to control diabetes due to the reasons of spending much time outside home, the desire to maintain independence, and the use of inappropriate coping styles such as avoidance [10, 11]. As a result of failing to control the disease, according to North American Nursing Diagnosis Association (NANDA) research and nursing diagnoses, adolescents suffer fatal cardiovascular complications, numerous psychological and behavioral problems, including depression, anxiety, and imposing exorbitant costs on the health and treatment system and families [12–18]. A non-medical way to control diabetes is empowerment through self-efficacy [19, 20]. Self-efficacy is defined as a person’s trust and confidence in their ability to perform a specific action [21, 22]. Chih et al. (2010), Rasbach et al. (2015) and Hosseini et al. (2014) reported a correlation between self-efficacy and blood sugar control [23–25]. Despite all the work done, poor to moderate self-efficacy has been reported [26–28]. Poor self-efficacy threatens patients with short-term and long-term physical and mental complications, thus they experience a lot of stress in their lives [3, 29]. Bandura, Barbaranelli, Caprara, and Pastorelli (1996) identified the pathways by which poor self-efficacy leads to social anxiety [15, 30]. Social anxiety is defined as the fear of social and functional situations [15, 31]. Children with diabetes have anxiety in their social interactions due to constant changes in their lives, fear of increased blood sugar and understanding of their differences with their peers [29]. Social anxiety leads to isolation from peers, non-compliance with treatment, improper blood sugar control, and creating progressive conditions for short-term and long-term complications of the disease [16, 32], and despite therapeutic interventions, we are still facing high prevalence, frequent relapses, and chronicity of social anxiety [33]. The World Health Organization has named health literacy as an influencing factor on the level of knowledge, the level of self-care and self-efficacy, and as a result more effective control and prevention of diabetes and its complications [3, 32–34]. Health literacy has been introduced as cognitive and social skills that determine the motivation and ability of people to acquire, understand and use information such that it leads to maintaining and improving their health and considered a key determinant of health and wellness at the population level [35–43]. It has different dimensions: functional (reading, comprehension, calculations), communicative (access, use, communication) and critical (evaluation, self-efficacy) [44]. The results of various studies have shown that in societies where the level of health literacy of the people is favorable, the people are cheerful and healthy and the governments are less likely to suffer exorbitant treatment costs. People with insufficient health literacy are less likely to understand the written and spoken information provided by health professionals and follow their instructions; they have a poorer health status, and incur more medical expenses [45]. Pourreza et al. (2012) showed that the health literacy of patients with diabetes in Tehran is poor [35].

Research in behavioral sciences needs a theory-oriented intervention [45, 46]. In this study, Bandura’s social learning theory (Bandura 1998) was used as the axis of intervention and teaching method [47–50]. Social learning theory assumes that people learn by observing others in a particular social group. All learning phenomena can practically happen through direct experience by observing the behavior of other people and the consequences of the behavior vicariously [51, 52]. This model has four stages: (1) attention, (2) retention, (3) re-creation, and (4) motivation. Observational learning models include live, verbal, and symbolic models [48, 53–55].

It should be noted that this research is in accordance with the seventh paragraph of the research priorities of the Children’s Department, School of Nursing and Midwifery, Tehran University of Medical Sciences, and the Ministry of Health (improving the health literacy of children, adolescents, and families). Also, health literacy education based on Bandura’s social learning theory, in order to improve the level of primary care, is used in the form of educational programs (prevention of infection and control of people at risk) according to the level of health literacy of people in primary care providers. And during the searches, they did not find an article with title " Social learning-based health literacy promotion on the self efficacy and social anxiety of adolescents with type 1 diabetes “. Therefore, this study aimed to “investigate the effect of implementing a health literacy training program based on Bandura’s social learning theory on self-efficacy and social anxiety of adolescents with T1DM”.

## Methods

### Study design and participants

This semi-experimental study was conducted with a pretest-posttest design and an intervention group and control group and 66 adolescents aged 15–18 years with T1DM (Iran, Tehran, 2022). Sampling was done by multi-stage random method as follows: in the first stage, two centers were randomly selected from among 7 endocrine and metabolism centers of Tehran province as the intervention and control groups. Endocrine and Metabolism Clinic of Children’s Medical Center Hospital (Government hospital affiliated to Tehran University of Medical Sciences) and Mofid Children’s Hospital Endocrine and Metabolism Clinic (Government hospital affiliated to Shahid Beheshti University of Medical Sciences) were selected as the control and intervention groups, respectively, randomly and by draw.And In the second stage, the participants were selected by Simple Random Sampling method (draw). In this way, separately for each group, the names of the qualified people who gave oral consent to participate in the study were put in a bag and 33 people were removed from the mixed name (In order not to transfer information between the control and intervention groups, the groups were selected separately from two different centers) and the intention to treat (ITT) method of analysis was used for the investigation.

The inclusion criteria were: aged 15–18 years old, living in Tehran, literate, able to speak and communicate, suffering T1DM based on the definite diagnosis recorded in the medical records, not having passed diabetes health literacy training courses, and access to smart electronic devices. And the exclusion criteria were: Unwillingness to participate in the study, not attending more than 2 sessions, not completing or incompletely completing the questionnaires.

The sample size was calculated as 66 individuals according to the studies and following formula, taking into account the maximum type I error of 5%, type II error of 20%, the standard deviation obtained for self-efficacy in the intervention group as 14.93 and in the control group as 17.99, and considering d = 15.25 [12]. Finally, 33 participants were recruited for each group considering 10% sample loss.

$$\mathrm\alpha=0.05=>\mathrm Z\;\left(1-\mathrm\alpha/2\right)\;=\;1.96$$

$$1-\mathrm\beta=\;0.2=>\mathrm Z\left(1-\mathrm\beta\right)\;=\;1.64$$

$$\mathrm\sigma1=\;14.93,\;\mathrm\sigma2=\;17.99$$

$$d=15.25n=\;\frac{2\left(Z_{(1-{\displaystyle\frac\alpha2}}+z_{1-\beta}\right)^2\left(\sigma1\ast\sigma2\right)}{d^2}=>n\;=\frac{2\left(1.96+1.64\right)^214.93\ast17.99}{2.62^2}=30$$

After receiving the code of ethics (IR.TUMS.FNM.REC.1399.038) from the Ethics Committee of the Faculty of Nursing, Midwifery and Rehabilitation of Tehran University of Medical Sciences, IRCT^1^ registration code (IRCT20210422051045N1) and making relevant arrangements, the researcher ran the validity and reliability tests for the questionnaires and designed the intervention. Thus, the content of the intervention was compiled by researchers using reliable sources and recent articles, Then the content of the intervention and the questionnaires were given to 10 expert professors in the field of children and adolescents and collected after 10 days. Finally, during a meeting, the researchers gathered and applied the comments, which mostly included improving the understanding of the sentences. And to check the reliability of the tools, test-retest and Pearson’s correlation coefficient methods were used. Pearson’s correlation coefficient scores for HELMA, SASA, and DMSES questionnaires were 0.85, 0.92, and 0.91, respectively. Then, an interview was conducted with adolescents in the endocrinology clinic of the Children’s Medical Center Hospital (control group) and the endocrinology clinic of Mofid Children’s Hospital (intervention group), and their parents were contacted if they were eligible to participate in the study. After briefing them on the research objectives and method, verbal and written consent was obtained from adolescents and parents(From 20 August, 2021 to 22 August, 2021). Before the start of the sessions, the questionnaires were given to the adolescents through social media(From 23 August, 2021 to 27 August, 2021) And the questionnaires were completed by adolescents with diabetes through self-report. Then, the day after collecting the questionnaires (taking the pretest), the training sessions were held in the form of seven training sessions of 30–45 min in a row during a week from 28 August, 2021 to 3 September, 2021 (in pre-arranged hours with adolescents). One month after the completion of the training sessions, the questionnaires were again given to the adolescents through social media [56–60], and collected after completion(4 October, 2021). The reason for choosing a one-month gap between the pre-test and the post-test was that most similar studies considered a gap of one month or less, and Professor Albert Bandura and his colleagues in their articles assessed the results of the tests as soon as possible, done on social learning theory (like the famous experiment on the Bobo doll). It should be noted that the implementation of the designed intervention was carried out under the supervision of supervisors and advisors, and the student, as a researcher, completed the course of communication with children and adolescents and the course for diabetes educators before starting the study and has a certificate. Virtual networks were used with the aim of improving informing and using the comments and suggestions of parents and adolescents. Throughout the intervention, adolescents who did not attend a session were contacted, and if they were absent for another session, they were excluded from the group. That, 1 person from the intervention group was excluded from the study due to non-responsiveness and two absences in training sessions (29,30 August). 1 other person from the intervention group was absent from the whole session due to the problem of accessing the internet and was therefore excluded from the study. Another 1 person from the intervention group was excluded from the study due to not completing the pre-test and post-test questionnaires. In the control group, 3 people were excluded from the study due to not completing the questionnaires.

### Implementing the intervention

The educational program was conducted such that each construct of diabetes health literacy was taught in one day, biut the use construct was taught in two days as it did not fit in a 45-minute session. According to the Bandura’s social learning theory, each of the health literacy constructs must go through these four stages for learning: attention, retention, re-creation, and motivation. To present the content in the first stage during each session, the attention of the audience was drawn to the subject (with the high knowledge of the presenter, stating the goals of the session, encouraging questions and answers to receive points). Furthermore, live and symbolic models were used to determine the manner of presenting the content based on the social learning theory, for instance, videos, pictures, PowerPoint presentations, speeches, problem solving, scenario execution, and Q&A were used to provide educational materials according to the contents. We used Skyroom™ so that the presenter’s image and voice could be seen and heard by the audience along with PowerPoint presentation and other video clips. After explaining the objectives, their attention was drawn to the contents and the present meeting. (The meeting was held in the Sky Room platform between the adolescents and the teacher in the form of a video conference). During the presentation, questions and answers were also held to motivate the adolescents to earn points. In the second stage (retention) according to Bandura’s social learning theory, the taught contents were imprinted in the learners’ mind as models and symbols. In the third stage (re-creation), the learned material was repeated and practiced to consolidate what was learned. Pre-prepared questions and scenarios (validation of educational content and questions were done by expert professors and fully explained in the method section) were asked to adolescents. In order to answer the questions, audio and video were available in the video conference. According to the pre-determined rules, they received points, and were thus motivated at this stage [30, 53, 55, 56]. The training program for promoting diabetes health literacy is shown in Table 1.

**Table 1:** Training program for promoting diabetes health literacy

Training sessions	Duration	The topic of the training session	Outlines	Teaching method	Evaluation
First	30-45 minutes	Access	Access to nutritional information, physical activity, insulin therapy, self-control of blood sugar and necessary follow-ups.	Interactive lectures, Q&A, problem solving, and exercises	Oral questions, doing exercises
Second	30-45 minutes	Use	Using nutritional information, self-control of blood sugar and necessary follow-ups.	Interactive lectures, Q&A, problem solving, and exercises	Oral questions, doing exercises
Third	30-45 minutes	Use	Use of physical activity information and insulin therapy	Interactive lectures, Q&A, problem solving, and exercises	Oral questions, doing exercises
Fourth	30-45 minutes	Calculation	calculation	Interactive lectures, Q&A, problem solving, and exercises	Oral questions, doing exercises
Fifth	30-45 minutes	Evaluation	Evaluation of nutritional information, physical activity, insulin therapy, self-control of blood sugar and necessary follow-ups.	Interactive lectures, Q&A, problem solving, and exercises	Oral questions, doing exercises
Sixth	30-45 minutes	Communication	Communication in the field of nutritional information, physical activity, insulin therapy, self-control of blood sugar and necessary follow-ups.	Interactive lectures, Q&A, problem solving, exercises, and scenario execution	Oral questions, doing exercises and scenario execution
Seventh	30-45 minutes	Self-efficacy	Self-efficacy in the fields of nutritional information, physical activity, insulin therapy, self-control of blood sugar and necessary follow-ups.	Interactive lectures, Q&A, problem solving, exercises, and scenario execution	Oral questions, doing exercises and scenario execution

Based on Bandura’s social-learning theory, learning and behavioral therapy are based on people’s understanding, and one of the most famous methods to create a better understanding for learning issues is using problem solving. Therefore, during the seven sessions, problem solving was used to improve the construct of understanding (among the constructs of health literacy) [21]. During all the sessions, the participating adolescents with T1DM were asked to read, repeat and practice the displayed materials and questions to improve their reading

Learning and behavioral therapy are based on people’s understanding, and one of the most famous methods to create a better understanding for learning issues is problem solving. Therefore, during the seven sessions, problem solving was used to improve the construct of understanding (among the constructs of health literacy) [21]. In all sessions, the adolescent participants with T1 diabetes were asked to read, repeat, and practice the displayed questions and texts. In this way, a step was taken to improve reading.

The course was game-based because the study “Motivational effect of games on education” by Andreas Hartmann in 2021 showed that the use of games in education creates intrinsic motivation for learning [61]. Game-centric means that adolescents earn points during the sessions to achieve certain rewards according to the following rules: (1) Each question answered correctly or scenario performed correctly is given a positive point. (2) In case of wrong answer to any question, no points were given and no points were deducted. (3) In case of correct answers to 5 consecutive questions, one point is added to the a adolescent’s score (4) Anyone who asked more questions to their friends would get an extra point at each stage, and finally, after the completion of the course, the top three people would receive a cash prize ($20 for the first person, $15 for each second person and $10 for the third person). The content taught to the intervention group was provided to the members of the intervention and control groups after the post-test.

### Data collection

The primary outcome of the current study is diabetes health literacy and secondary outcomes are functional self-efficacy and social anxiety. Data were collected using four questionnaires; (A) Demographic Information Questionnaire, (B) Health Literacy Measure for Adolescents (HELMA), (C) Social Anxiety Scale for Adolescents (SASA) by Puklek, (D) Diabetes Mellitus Self-Efficacy Scale (DMSES).

A) Demographic information questionnaire had 36 items related to demographic characteristics, which was validated by expert professors.
B) Health Literacy Measure for Adolescents (HELMA) has 44 items in eight dimensions of access, reading, comprehension, evaluation, use, communication, self-efficacy and calculation that are related to public health literacy. The researcher, along with expert professors in this field and using reliable scientific sources, moved the concept of items towards the examination of T1DM health literacy. There was no change made in the number of items in each field. Items are scored based on a five-point Likert scale from 1 (never) to 5 (always). The minimum and maximum scores of health literacy are 0 and 100, which, based on the cut-off points of 50, 66 and 84, divide adolescents’ health literacy into four levels of inadequate (0–50), not very adequate (50.1–66), favorable (66.1–84) and excellent (84.1–100) [38].
C) Social Anxiety Scale for Adolescents (SASA) by Puklek has 28 items and two subscales of apprehension and fear of negative evaluation - AFNE and tension and inhibition in social contact - TISC. It is scored based on five-point scale from 1 (totally disagree) to 5 (totally agree). The minimum and maximum scores are 28 and 140, respectively, where the score ranges of 28–46, 46–93 and 93–140 indicate low, moderate and high social anxiety, respectively [62, 63].
D) Diabetes Mellitus Self-Efficacy Scale (DMSES) has 19 items scored based on a Likert scale from “I can’t at all” to “I definitely can”. The total score range is 0-199, and the score ranges 0–66, 66–130 and 130–199 indicate low, moderate and high self-efficacy, respectively [21, 64].

### Data analysis

Collected data were analysed in SPSS-25 software using descriptive statistics, analytical statistics (chi-square, paired t-test, ANCOVA, correlation coefficients, independent t, and Fisher’s exact test) and Kolmogorov-Smirnov test to check the normality of data distribution. The significance of the findings was considered (*P* < 0.001). Due to the high importance of the issue of health literacy and other variables of the study, with the opinion of the research team and the statistical consultant, the significance level of (*P* < 0.001) was considered.

### Findings

#### Demographic characteristics

The intervention and control groups were statistically matched in terms of background and confounding variables that could affect the results of the research. Normality of variables of health literacy, self-efficacy and social anxiety of adolescents with T1DM was assessed, and the results of the Kolmogorov-Smirnov test showed that all the studied variables were normal (*P* < 0.05). Therefore, parametric tests were used to investigate the objectives of the study.

In general, these people did not consider diabetes as a limitation and mostly spent their time on the Internet, but they did not know how to get the information they needed at different times and places. They had a desire to learn, but because they had attended many classes but their self-efficacy had not improved, they did not show a desire to participate in the study at first. According to them, the obstacles to improving self-efficacy were: the inability to participate in training sessions due to the long distance, the inability to properly understand the intended content in the sessions due to the use of specialized terms, lack of training SMBG^2^ and glycemic index calculations in Educational classes, non-participation in educational classes due to parents being too busy (not accompanying parents in educational classes), not having enough information on diabetes, its complications and control.

#### Investigating the effect of educational intervention on diabetes health literacy

The results of the independent t-test showed that the level of health literacy and all its dimensions did not have a significant difference between the two groups (*P* > 0.05) at the baseline, while this difference was significant (*P* < 0.001) after the intervention, that is, the scores of the intervention group were significantly higher than those of the control group (Table 2). Also, independent t-test indicated that the increase in health literacy scores after the study was significantly higher in the intervention group (25.90 ± 9.53) than in the control group (2.06 ± 5.11) (*P* < 0.001), and the increase in health literacy dimensions was significantly higher in the intervention group than in the control group (*P* < 0.001) (Table 3). As shown in Table 2, the effect size is greater than 0.08, which means that the intervention had a high effect on health literacy and its dimensions. The largest effect size was in the dimension of communication and the smallest in the dimension of calculations.

**Table 2:** Comparison of the level of health literacy and its dimensions in adolescents with T1DM in the intervention and control groups before and after the intervention

Group	Time of intervention	Intervention		*P* Value	Interpretation	Control		*P* Value	Interpretation	Result		
Health literacy and its dimensions (0-100)		Mean	SD			Mean	SD					
Access	Before	32.70	10.59	*P* > 0/001	inadequate	33.95	7.64	*P* = 0/522	inadequate	*P* = 0.602	df = 58	t^a^ = 0.524
	After	57.08	4.85		not very adequate	34.58	9.67		inadequate	*P* < 0.001	F^b^ = 167.495	Partial Eta Squared^c^=0.746
Reading	Before	27.66	12.15	*P* > 0/001	inadequate	23.33	8.64	*P* = 0/544	inadequate	*P* = 0.117	df = 58	t = -1.591
	After	53.66	6.68		not very adequate	21.33	16.65		inadequate	*P* < 0.001	F = 90.460	Partial Eta Squared=0.613
Understanding	Before	62.83	14.48	*P* > 0/001	not very adequate	63.50	11.38	*P* = 0/420	not very adequate	*P* = 0.844	df = 58	t = 0.198
	After	89.0	6.46		excellent	64.33	11.19		not very adequate	*P* < 0.001	F = 165.741	Partial Eta Squared=0.744
Evaluation	Before	71.58	9.41	*P* > 0/001	favorable	68.91	10.72	*P* = 0/300	favorable	*P* = 0.310	df = 58	t = -1.024
	After	85.25	5.81		excellent	69.83	9.42		favorable	*P* < 0.001	F = 142.620	Partial Eta Squared=0.714
Use	Before	40.66	8.78	*P* > 0/001	inadequate	73.33	10.80	*P* = 0/363	favorable	*P* = 0.195	df = 58	t = -1.311
	After	68.33	9.31		favorable	38.50	12.04		inadequate	*P* < 0.001	F = 192.924	Partial Eta Squared=0.772
Communication	Before	38.12	11.05	*P* > 0/001	inadequate	40.41	9.24	*P* = 0/095	inadequate	*P* = 0.387	df = 58	t = 0.871
	After	77.70	9.52	*P* > 0/001	favorable	42.29	10.33		inadequate	*P* < 0.001	F = 371.661	Partial Eta Squared=0.867
Self-efficacy	Before	72.70	9.46	*P* > 0/001	favorable	68.54	15.72	*P* = 0/142	favorable	*P* = 0.219	df = 58	t = -1.244
	After	84.37	4.64		excellent	69.47	14.90		favorable	*P* < 0.001	F = 62.721	Partial Eta Squared=0.524
Calculation	Before	30.51	10.0	*P* > 0/001	inadequate	34.57	13.33	*P* = 0/161	inadequate	*P* = 0.694	df = 58	t = 0.396
	After	50.74	46.66		not very adequate	40.68	20.0		inadequate	*P* < 0.001	F = 8.559	Partial Eta Squared=0.131
Health literacy level (0-100)	Before	49.31	6.10	*P* > 0/001	Inadequate	48.67	7.28	*P* = 0/055	Inadequate	*P* = 0.711	df = 58	t = 0.372
	After	75.22	8.38		Favorable	50.73	7.72		Not very adequate	*P* < 0.001	F = 170.270	Partial Eta Squared=0.749

^a^T-Test

^b^ANCOVA (Analysis of Covariance)

^c^Effect size

**Table 3:** Numerical indices of changes in the level of health literacy and its dimensions in adolescents with T1DM in the control and intervention groups and comparing the means

Group	Control		Intervention		Independent t-test results		
	Mean	SD	Mean	SD			
Health literacy and its dimensions							
Self-efficacy	0.62	5.28	24.37	11.41	*P* < 0.001	df = 58	t = -10.344
Access	-2.0	17.84	26.0	14.16	*P* < 0.001	df = 58	t = -6.732
Reading	0.83	5.58	26.16	14.06	*P* < 0.001	df = 58	t = -9.171
Understanding	0.91	4.75	13.66	6.42	*P* < 0.001	df = 58	t = -8.737
Evaluation	1.16	6.90	27.66	8.38	*P* < 0.001	df = 58	t = -13.364
Use	1.87	5.95	39.58	9.75	*P* < 0.001	df = 58	t = -18.072
Communication	0.93	3.39	11.66	8.79	*P* < 0.001	df = 58	t = -6.234
Calculation	6.66	25.37	36.66	49.01	*P* < 0.001	df = 58	t = -2.977
Health literacy level	2.06	5.11	25.90	9.53	*P* = 0.004	df = 58	t = -12.069

#### Investigating the effect of educational intervention on self-efficacy

The results of the independent t-test showed that self-efficacy and all its dimensions did not have a significant difference between the two groups (*P* > 0.05) at the baseline, while this difference was significant (*P* < 0.001) after the intervention, that is, the scores of the intervention group was significantly higher than the control group (Table 4). The independent t-test results also showed that the self-efficacy scores increased significantly in the intervention group (26.16 ± 11.26) compared to the control group (1.76 ± 7.30) (*P* < 0.001). Also, the findings showed that the increase in self-efficacy scores was significantly higher in the intervention group than in the control group (*P* < 0.05) (Table 5). As shown in Table 4, the effect size is greater than 0.08, which means that the intervention had a high effect on self-efficacy and its dimensions. The largest effect size was in the dimension of physical activity and weight control, and the smallest was in the dimension of insulin therapy.

**Table 4:** Comparison of self-efficacy and its dimensions in adolescents with T1DM in the intervention and control groups before and after the intervention

Group	Time of intervention	Intervention		*P* value	Interpretation	Control		*P* value	Interpretation	Results		
Self-efficacy and dimensions		Mean	SD			Mean	SD					
Blood sugar measurement (0-30)	Before	26.16	1.48	*P* > 0/001	High	22.90	2.66	*P* < 0.357	High	*P* = 635	df = 58	t = -0.478
	After	27.0	2.22		High	22.33	3.17		High	*P* < 0.001	F = 42.90	Partial Eta Squared=0.429
Physical activity and weight control (0-40)	Before	27.26	1.94	*P* > 0/001	High	26.30	3.86	*P* < 0.625	Moderate	*P* = 227	df = 58	t = -1.222
	After	32.56	2.09		High	26.36	3.77		Moderate	*P* < 0.001	F = 128.625	Partial Eta Squared=0.693
Screening (0-20)	Before	13.76	3.03	*P* > 0/001	Moderate	15.20	3.43	*P* < 0.103	High	*P* = 092	df = 58	t = 1.712
	After	16.83	1.28		High	15.33	3.47		High	*P* < 0.001	F = 20.667	Partial Eta Squared=0.266
Insulin therapy (0-20)	Before	14.26	2.58	*P* > 0/001	Moderate	14.20	1.76	*P* < 0.573	Moderate	*P* = 908	df = 58	t = -0.117
	After	16.10	2.77		High	14.13	1.87		Moderate	*P* < 0.001	F = 10.491	Partial Eta Squared=0.155
Diet (0-80)	Before	46.66	8.96	*P* > 0/001	Moderate	43.83	9.82	*P* < 0.060	Moderate	*P* = 248	df = 58	t = -1.167
	After	58.80	8.64		High	46.03	13.14		Moderate	*P* < 0.001	F = 25.976	Partial Eta Squared=0.313
Self-efficacy (0-190)	Before	125.13	10.41	*P* > 0/001	Moderate	122.43	14.35	*P* < 0.196	Moderate	*P* = 408	df = 58	t = -0.834
	After	151.30	10.71		High	142.20	17.67		Moderate	*P* < 0.001	F = 100.488	Partial Eta Squared=0.638

**Table 5:** Comparison of changes in self-efficacy of adolescents with T1DM in intervention and control groups and comparison of means

Group	Control		Intervention		Independent t-test results		
Self-efficacy and dimensions	Mean	Standard deviation	Mean	SD			
Blood sugar measurement	-0.56	3.31	3.83	2.96	*P* < 0.001	df = 58	t = -5.419
Physical activity and weight control	0.06	0.73	5.30	2.60	*P* < 0.001	df = 58	t = -10.597
Screening	0.13	0.43	3.06	3.33	*P* < 0.001	df = 58	t = -4.782
Insulin therapy	-0.06	0.63	1.83	4.03	*P* = 0.014	df = 58	t = -2.547
Diet	-3.93	7.08	5.90	8.11	*P* < 0.001	df = 58	t = 5.0
Self-efficacy	1.76	7.30	26.16	11.26	*P* < 0.001	df = 58	t = -9.950

#### Investigating the effect of educational intervention on social anxiety

The results of the independent t-test showed that the social anxiety and all its dimensions did not have a significant difference between the two groups (*P* > 0.05) at the baseline, while this difference was significant (*P* < 0.001) after the intervention, that is, the scores of the intervention group was significantly lower than the control group (Table 6). Also, using independent t-test, it was reported that the increase in social anxiety scores after the study was significantly lower in the intervention group (-8.33 ± 7.16) than in the control group (-0.10 ± 3.78) (*P* < 0.001); and the decrease in the scores of social anxiety dimensions was significantly more in the intervention group than in the control group (*P* < 0.001) (Table 7). As shown in Table 6, the effect size is greater than 0.08, which means that the intervention had a high effect on social anxiety and its dimensions. The largest effect size was in the dimension of social anxiety and the smallest was in the dimension of tension and inhibition in social encounters.

**Table 6:** Comparison of social anxiety and its dimensions in adolescents with T1DM in intervention and control groups before and after the study

Group	Time of intervention	Intervention		*P* value	Interpretation	Control		*P* value	Interpretation	Independent t-test results		
Social-anxiety and dimensions		Mean	SD			Mean	SD					
Apprehension and Fear of Negative Evaluation (15-75)	Before	58.63	2.64	*P* > 0/001	High	59.80	4.23	*P* > 0/886	High	*P* = 0.979	df = 58	t = 0.026
	After	54.03	3.24		Moderate	59.63	4.37		High	*P* < 0.001	F = 29.218	Partial Eta Squared=0.339
Tension and Inhibition in Social Contact (13-65)	Before	39.83	2.78	*P* > 0/001	Moderate	38.63	3.04	*P* > 0/783	Moderate	*P* = 0.116	df = 58	t = -1.594
	After	36.10	2.86		Moderate	38.70	3.91		Moderate	*P* < 0.001	F = 22.797	Partial Eta Squared=0.286
Social anxiety (28-140)	Before	98.47	3.91	*P* > 0/001	High	98.43	5.83	*P* > 0/839	High	*P* < 0.001	df = 58	t = 5.633
	After	90.13	4.66		Moderate	98.33	6.16		High	*P* < 0.001	F = 39.6554	Partial Eta Squared=0.410

**Table 7:** Comparison of changes in social anxiety of adolescents with T1DM in intervention and control groups and comparison of means

Group	Control		Intervention		Independent t-test results		
	Mean	SD	Mean	SD			
Social anxiety and dimensions							
Apprehension and fear of negative evaluation	-0.17	3.28	-4.60	5.02	*P* < 0.001	df =	t = -3.878
Tension and inhibition in social contact	0.07	1.78	-3.73	3.61	*P* < 0.001	df =	t = -3.878
Social anxiety	-0.10	3.78	-8.33	7.16	*P* < 0.001	df =	t = -3.878

**Fig. 1:**
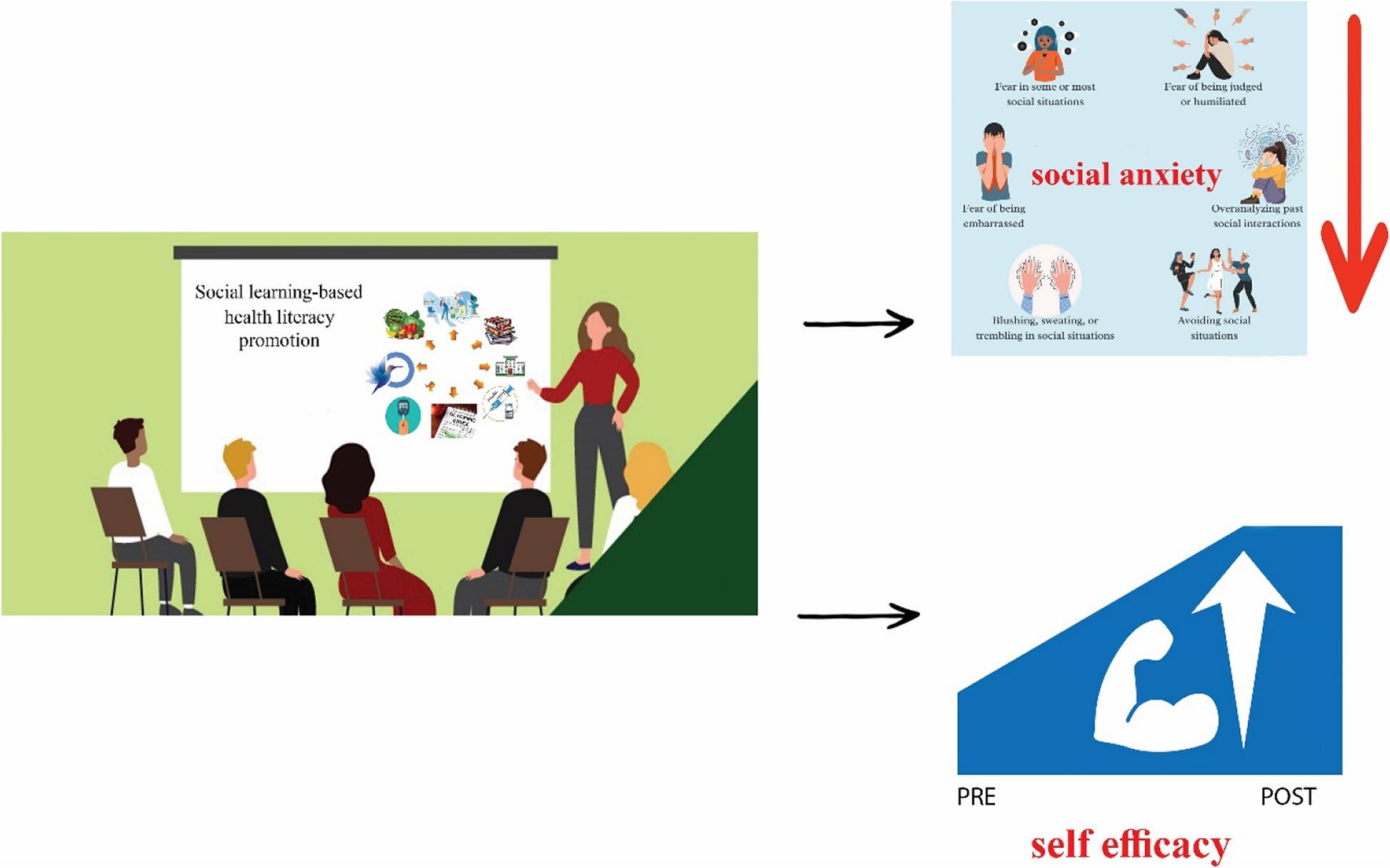
Summary of findings

The summary of the findings is depicted in the figure below (Fig. 1).

## Discussion

According to the results of the study, recommended to hold diabetes health literacy promotion workshops in health education centers to improve the management of diabetes, reduce the physical-psychological complications and the financial burden on the individual, the family and the healthcare system.

As reported, the two groups were statistically matched in terms of background and confounding variables that could affect the results of the research, so it can be concluded that the results obtained by comparing the scores in the intervention and control groups were due to the implementation of the intervention.

The current research reported the diabetes health literacy in the intervention group turned from insufficient before the study to favorable after the intervention. Banca et al. (2019) and Huang et al. (2021) noted the positive impact of health literacy education in four areas (nutrition, insulin therapy, exercise, and self-control of blood sugar) through games and creating motivation [65, 66]. The results can be explained a adolescents with T1DM need to receive this knowledge in four main areas due to their age and the significant role of health literacy in their knowledge to correctly manage the disease. Learning requires motivation. Therefore, a motivational game was used, and because behavioral therapy requires theory-based intervention, Bandura’s social learning theory was used in health literacy education [45, 46]. The basis of the tools used in all health literacy improvement studies, like the present study, is the general health literacy tool, which has been modified according to the type of variable studied in the research, and the necessary validity and reliability checks have been done.

The results of the present study reported the self-efficacy score of the intervention group before and after the study as poor and moderate, respectively. Medina et al. (2022) found no significant relationship between functional health literacy and self-efficacy in diabetes, and they also reported moderate functional health literacy and high self-efficacy [67]. However, studies by Shahbazi et al. (2018), Rafizadeh et al. (2015), Zareipour et al. (2021), and Mszarei et al. (2021) found a positive and significant correlation between self-efficacy and overall health literacy and its components, and showed that as people’s health literacy increases, their self-efficacy also increases [68–71]. Their results are consistent with the present study. The review of inconsistent studies shows the lack of use of the specific theoretical framework of empowerment, the lack of attention to all aspects of empowerment, the neglect of the effect of demographic variables on the empowerment of patients, and the lack of program follow-up.

Surveys indicate high and moderate scores for social anxiety in the intervention group before and after the study, respectively. Soltanizadeh et al. (2019) reported a significant difference in the mean scores of social anxiety dimensions between the two groups before and after the intervention [72]. Tang et al. (2022) showed that social anxiety is higher in the group of adolescents with T1DM than in the peer group without T1DM [73]. Rechenberg et al. suggested that anxiety is common among adolescents with type 1 diabetes and is directly related to increased HbA1c, poor self-care, depressive symptoms, fear of hypoglycemia, and poor blood sugar control [74]. The studies found by the researcher were consistent with the results of the present study and study with different results were not found [72–74]. Adolescents with T1DM feel different from other peers due to the chronic illness, insulin injections, signs and of increased or decreased blood sugar, and this makes them feel uncomfortable and anxious in the company of their peers. Health literacy has six domains (access, use, calculation, evaluation, communication and self-efficacy), which according to the education program, the promotion of diabetes health literacy led to the improvement of those areas and the creation of self-efficacy in diabetes. Therefore, by creating self-efficacy in diabetes and learning diabetes management, adolescents learn how to live with their disease in the best way and not to feel different from peers.

Therefore, considering the clarification of the effect of health literacy behavioral intervention on the functional self-efficacy and social anxiety of teenagers with type 1 diabetes, we expect to investigate and improve the health literacy of teenagers with type 1 diabetes (using this plan) in clinics and hospitals. lead to a reduction in visits to the emergency room and hospitals, a reduction in short-term and long-term complications in affected people, a reduction in absenteeism from school, and a reduction in fear and social anxiety, and on the other hand, a heavy financial burden on the Ministry of Health, society, reduce the family and the individual. Also, increasing the self-efficacy of patients will lead to a decrease in hospitalization rates and eventually decrease the transmission of nosocomial infections. And also by reducing social anxiety, we will lead to an increase in patients’ self-confidence and self-esteem [23–26, 32–34, 41].

## Conclusion

In conclusion, this research showed that health literacy training is effective in improving self-efficacy and reducing social anxiety among adolescents with T1DM. Therefore, it is suggested to evaluate patients’ level of health literacy in health and treatment systems before providing them with information, and customize information provision according to their level of health literacy. The reason is that if health experts provide the necessary information and training according to individual’s level of health literacy, it will be better understood and more effective. Furthermore, considering the abundance of people suffering from diabetes in the world and in Iran, based on the studies, the chronicity of diabetes and the lack of a definitive treatment for diabetes, it is possible to spend most of the healthcare costs on preventing complications and disabilities by improving the health literacy of adolescents with T1DM and as a result better control of blood sugar. Increased self-efficacy and reduced social anxiety in adolescents can reduce physical, mental and behavioral complications caused by diabetes on the individual, family, society and healthcare system. Finally, the results of this research can be used to improve the capabilities of adolescents with T1DM and develop operational-educational programs in the centers where these adolescents receive various services. Also, health literacy education, in order to improve the level of primary care, is used in the form of educational programs (prevention of infection and control of people at risk) according to the level of health literacy of people in primary care providers.

### Limitations

Since this research was conducted during the COVID-19 pandemic and the participants were at a higher risk, fewer adolescents with T1DM came to the endocrinology and diabetes clinics, therefore, the sampling process was slow. Furthermore, adolescents had many school and sports programs during the day and could hardly attend classes. Other limitations in the current study were the inability to hold face-to-face meetings due to the covid-19 pandemic and Low motivation of adolescents to participate in the training course. There were also financial constraints and the prizes awarded to the adolescents were funded by the author.

The strength of the current study was the use of social learning theory to promote diabetes health literacy.

Although neighborhood environments significantly affect the development of diabetes risk factors, complications and mortality during people’s lives, because adolescents with type 1 diabetes are almost all in terms of living environment and access to facilities and equipment were homogeneous, in the present study we were not able to investigate the impact of neighborhood environments on the risk factors and complications of diabetes.

### Recommendations

It is recommended to investigate the demographic factors related to health literacy, self-efficacy and social anxiety of adolescents with T1DM in future studies. similar studies with larger sample sizes (for generalization) should be conducted in other cities and endocrinology and diabetes clinics (to investigate the effect of neighborhood environments on the incidence of diabetes and its complications), and the results should be compared with the results of the present study. It’s also recommended to investigate the effect of improving health literacy on diabetes indicators, including: HbA1c, 2hpp, FBS. Screening for cardiovascular disease risk factors in an urban low-income. using social learning theory to explore the process of learning from role models in clinical settings. Cognitive and social learning models of drug dependence: implications for the assessment of tobacco dependence in adolescents. The use of social cognitive learning for humanistic professional role modelling: impacts on awareness of humanistic professionalism, caring behaviour, and transitional anxiety. Investigating whether universal screening for gestational diabetes improves neonatal outcomes in a socially vulnerable population?

## Data Availability

The excel data used to support the findings of this study are available from the corresponding author upon request.

## Footnotes

1 Iranian Registry of Clinical Trials.

2 Self-monitoring Blood Glucose.
